# Expanded Perlite-Reinforced Alginate Xerogels: A Chemical Approach to Sustainable Building and Packaging Materials

**DOI:** 10.3390/gels10120782

**Published:** 2024-11-29

**Authors:** Radmila Damjanović, Marija M. Vuksanović, Miloš Petrović, Željko Radovanović, Milena Stavrić, Radmila Jančić Heinemann, Irena Živković

**Affiliations:** 1 Faculty of Technology and Metallurgy, University of Belgrade, 11000 Belgrade, Serbia; radabdamjanovic@gmail.com (R.D.); mpetrovic@tmf.bg.ac.rs (M.P.); and radica@tmf.bg.ac.rs (R.J.H.); 2 VINČA Institute of Nuclear Sciences—National Institute of the Republic of Serbia, University of Belgrade, 11351 Belgrade, Serbia; 3 Innovation Centre of Faculty of Technology and Metallurgy doo, 11000 Belgrade, Serbia; zradovanovic@tmf.bg.ac.rs; 4 Institute of Architecture and Media, Graz University of Technology, 8010 Graz, Austria; mstavric@tugraz.at; 5 Faculty of Applied Arts, University of Arts in Belgrade, 11000 Belgrade, Serbia; irena.zivkovic@fpu.bg.ac.rs

**Keywords:** alginate, xerogel, expanded perlite, biocomposite, Brazilian test, elastic modulus

## Abstract

In sustainable construction and packaging, the development of novel bio-based materials is crucial, driving a re-evaluation of traditional components. Lightweight, biodegradable materials, including xerogels, have great potential in architectural and packaging applications. However, reinforcing these materials to improve their mechanical strength remains a challenge. Alginate is a promising matrix material that may be compatible with inorganic fibrous or particulate materials. In this study, biocomposite xerogel-structured foam materials based on an alginate matrix with expanded perlite reinforcement are improved using certain additives in different weight ratios. The plasticizers used include glycerol and gum arabic, while chitosan was added as an additional reinforcement, and iota carrageenan was added as a stabilizer. The tested specimens, with varying weight ratios of the added components, showed good mechanical behavior that highlights their potential use as packaging and/or architectural materials. The influence of the presence of different components in the composite material specimens on the modulus of elasticity was investigated using SEM images and FTIR analyses of the specimens. The results show that the specimen with the largest improvement in the elastic modulus contained a combination of chitosan and glycerol at a lower percentage (1.96 MPa), and the specimen with the largest improvement in tensile strength was the specimen containing chitosan with no plasticizers (120 kPa), compared to cases where combinations of other materials are present.

## 1. Introduction

**Figure 1:**
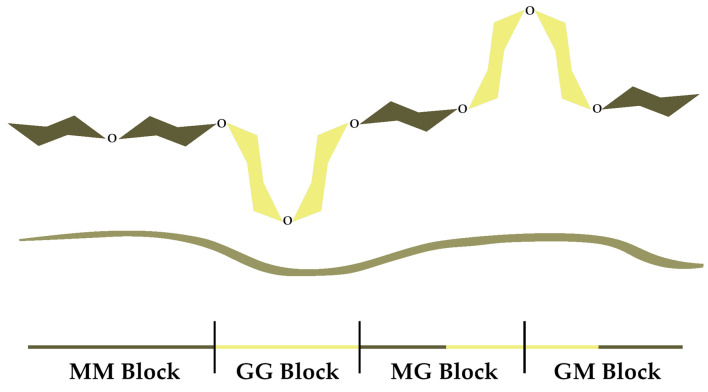
Schematic structure of alginate.

Alginate, a polysaccharide found in brown algae, is a linear copolymer consisting of residues of α-L-guluronic (G) and β-D-mannuronic (M) acids that are connected by 1,4 glycosidic bonds, where the G and M sequences differ in terms of number and position (Figure 1) [1].

Alginates are generally available in the form of sodium, potassium, or ammonium salts [2]. These monovalent ions containing salts are water-soluble, whilst alginate salts of many bivalent cations, such as Ca^2+^, Sr^2+^, and Ba^2+^, are water-insoluble [3]. The gelling property of alginates, in addition to other favorable properties such as their relatively low cost, strong bioadhesion and absorption, ecofriendliness, non-toxicity, biocompatibility, and biocompostability [4], enables their usage in many different fields [5].

**Figure 2:**
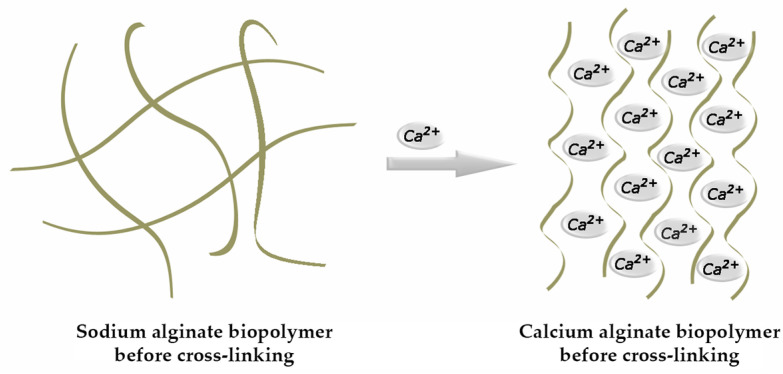
Formation of an egg-box structure.

The above-mentioned gel-forming ability is considered its supreme functional property. Of the cations used to synthesize alginate ionic gel, the Ca^2+^ ion is the most commonly used [6]. Gelation occurs through its binding with G sections of the alginate molecule, during which a specific “egg-box” configuration is formed (Figure 2) [7].

Alginate has a broad range of applications; it is used in the food and packaging industry [4], biomedical sciences and engineering [8,9], the pharmaceutical industry [10], dentistry [11], wastewater treatment [12], and textile printing [13]. Apart from this, it is also used as a flame retardant and insulation material [14,15,16]. Alginate materials are used in the form of film, beads, capsules, fibers, sponges, foams, hydrogels, and 3D-printed matrices [17,18]. The disadvantages of using alginate without other components lie in its low stability and poor mechanical properties [4]. Its properties can be improved by adding plasticizers [19,20] and reinforcements, which may be in the form of nanofibers, nanoparticles, nanotubes, and other polymers [5]; fibers [21]; or crop byproducts [22,23].

The addition of plasticizers to biopolymers increases their flexibility and processability [24] and reduces the shrinking and brittleness of polymers [25]. One of the most commonly added plasticizers in alginate is glycerol [19], a polyol that due to the presence of hydroxyl groups can form hydrogen bonds with alginate polymer chains, thus reducing the hydrogen bonding between them [26]. It has been reported that it enhances the moisture content, elongation at break, and flexibility of alginate films [24,25,27]. It is also a byproduct of biodiesel green technology production [28], and it is non-toxic and biodegradable [29]; therefore, it can be used to meet the global demand for the production of materials using renewable resources.

Naturally occurring gum arabic, which is a non-toxic and biodegradable anionic polysaccharide [30,31] obtained from the acacia tree, is known for its role as a stabilizer, emulsifier, and thickener, and has also been reported to improve alginate properties [31,32]. Additionally, it has been used as a plasticizer in construction materials [33]. In combination with alginate, it also displays favorable adhesion properties [34].

Chitosan, a non-toxic and biodegradable [35] cationic polysaccharide derived from crustacean skeletons [36], can also be combined with alginate. Studies have found that it improves mechanical properties of alginate hydrogels, such as Young’s modulus and stiffness [37] and mechanical resistance [38,39].

Carrageenan, a sulfated linear polysaccharide obtained from red algae, has been used as a stabilizing, thickening, and gelling agent. One of the most commonly used carrageenan types is iota carrageenan [40], which is biodegradable and forms soft, elastic, and cohesive gels [41].

Perlite is an amorphous alumino-silicate volcanic rock, with general composition given as SiO_2_ and Al_2_O_3_ content, and their average range values of 70–75% and 10–15%, respectively [42,43]. When heated to 900–1200 °C, it expands, and the volume is increased by five- to twenty-fold [44]. Due to the evaporation of water entrapped within its inner perlite layers, expanded perlite has a specific honeycomb microstructure and is lightweight, which makes it favorable for usage as a filler [42]. Expanded perlite possesses a low thermal conductivity and high sound absorption; it is also fire-resistant, has a low bulk density, and is used in the following fields: the construction industry; thermal, sound, and cryogenic insulation; agriculture; and as an adsorbent material and filter medium [45,46]. It is environmentally friendly and has been used as a component of various composite materials in buildings [47,48].

Upon reviewing the data, several studies were found to have explicitly combined alginate and perlite for use as an aerogel photothermal conversion material [49], as composite particles for ion removal [50], as beads for dye adsorbents [51], as encapsulation microbeads [52], and as encapsulation for perlite/alginate carbon nanotubes [53].

This study is a novel investigation into alginate-expanded perlite materials in the form of foam, as no prior research on this specific combination of components in this form was found in the existing literature. To explore this novel material further, 24 distinct composite formulations were investigated, each consisting of an alginate matrix, expanded perlite reinforcement, and a unique combination of the previously mentioned additives. Four of these formulations were prepared using a synthetic acrylic binder (plasticizer) for comparison purposes. The specimens were prepared using a newly proposed foaming procedure and were subjected to testing according to the Brazilian test, which is used for the indirect measurements of the tensile strength of brittle materials [54,55,56,57,58,59,60,61,62,63].

In this paper, new ecofriendly and biodegradable biocomposite foam materials are proposed that could be used as packaging or architectural materials. They pose no threat to the environment and can be disposed of in the ground, playing a role in plant nutrition and growth. The materials are based on alginate matrices and expanded perlite reinforcement, which have not yet been reported in the available literature in foam form. All components added to the composite, except the acrylic binder which was used to prepare the comparison group of specimens, are biobased, ecofriendly, and biodegradable. The aim of this study is to investigate the influence of the presence of different biobased additives in biocomposite materials using the obtained mechanical testing data.

## 2. Results and Discussion

### 2.1. Image Analysis

**Figure 3:**
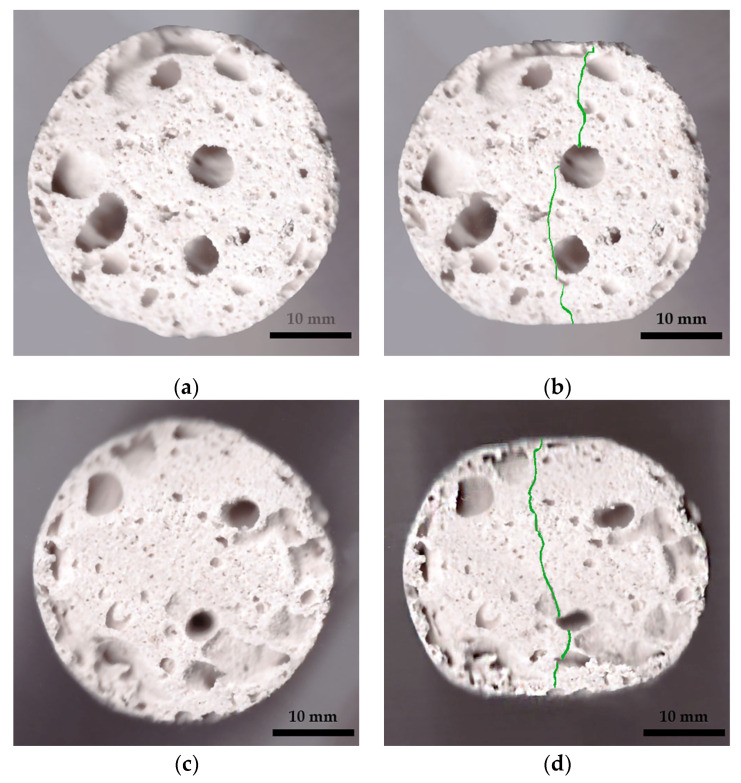
Examples of specimen shapes before and after the Brazilian test. Specimen 0 (A+P): (**a**) before testing and (**b**) after testing, with the crack line marked; Specimen 8 (A+P+15Gl): (**c**) before testing and (**d**) after testing, with the crack line marked.

**Figure 4:**
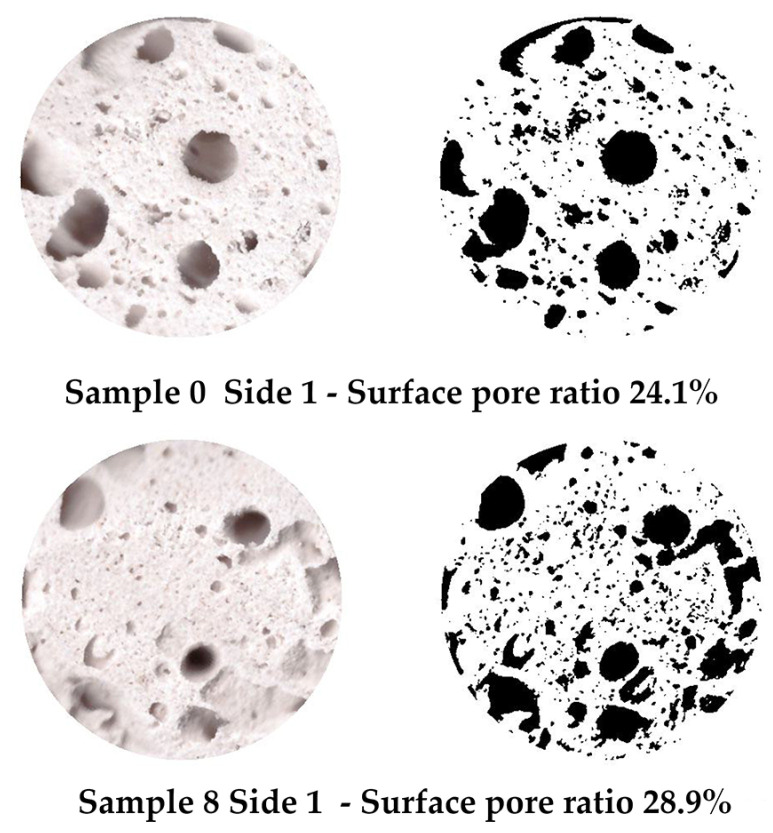
Examples of labeled surface pores on Specimen 0 (A+P) and Specimen 8 (A+P+15Gl) and calculated surface pore ratios.

**Figure 5:**
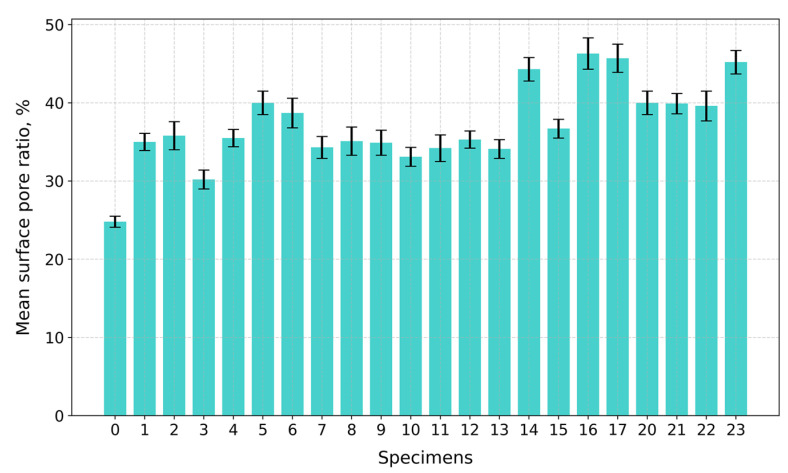
Mean surface pore ratios of tested specimens.

**Figure 6:**
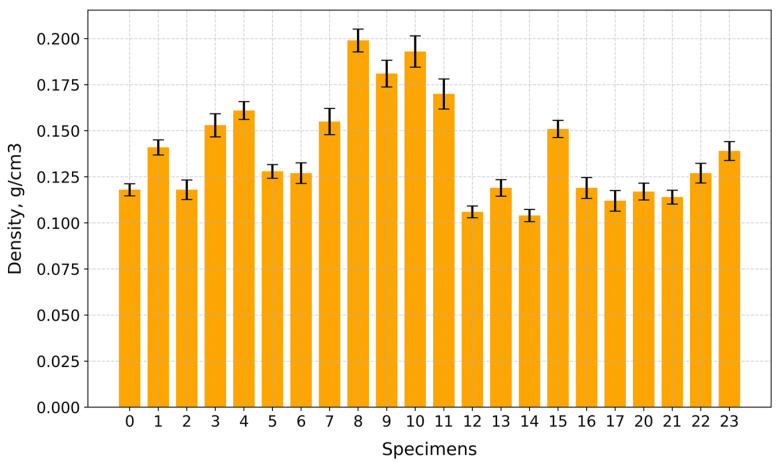
Density values of tested specimens.

**Figure 7:**
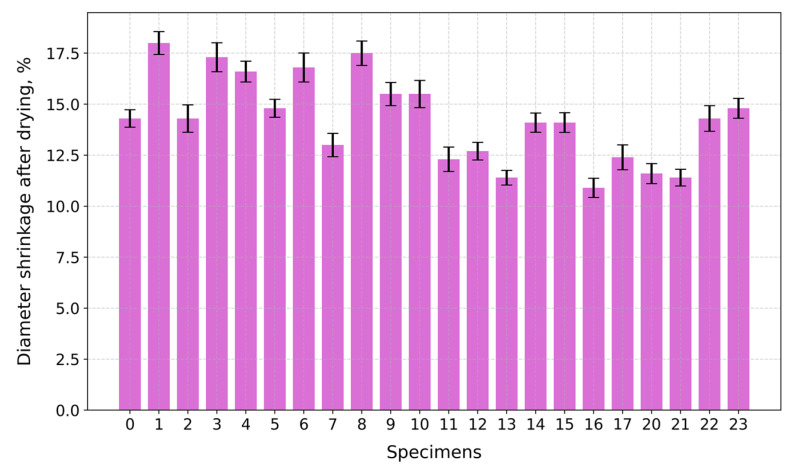
Diameter shrinkage of tested specimens after drying.

Under compression, the surface at the top and bottom contact points of the disk-shaped specimens (Figure 3a,c) underwent flattening, forming load and crack lines (Figure 3b,d) stretching from the top to the bottom of the flattened load contact surfaces. For the sake of uniformity, all specimen images were processed and centrally cropped to the same diameter and then segmented to determine the areas of pores on the specimen surfaces and their ratios (Figure 4). This behavior was observed for all specimens, regardless of their composition. The mean surface pore ratios (the mean value of the pore ratio on both sides of specimen) fall within the range of 24.8–46.3% (Figure 5), while the density values circle around 0.104–0.199 g/cm^3^ (Figure 6), and the diameter shrinkage, after drying, is in the range of 11.4–18.0% (Figure 7). These values are presented in Table 1.

**Figure 8:**
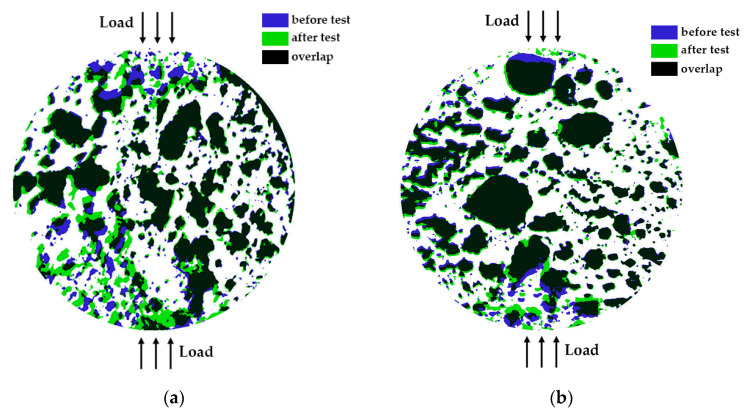
Visual overview of surface pore shape changes for selected specimens: (**a**) Specimen 5 (A+P+Ch+5Gl), and (**b**) Specimen 12 (A+P+1GA) after testing.

Figure 8 displays the change in the specimens’ pore shapes after testing. As can be observed, there are no visible shape alterations for the largest pores, except when these large pores are close to the loading areas and are within the plastic deformation zone. Small pores, however, undergo shape changes in the near-load regions and even in the central areas for some specimens.

**Figure 9:**
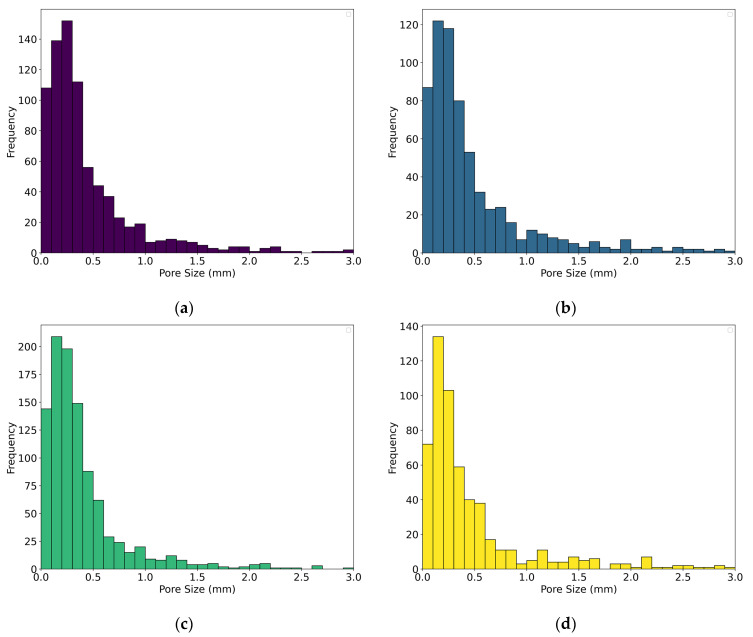
Pore size distribution chart for (**a**) Specimen 0 (A+P), (**b**) Specimen 5 (A+P+Ch+5Gl), (**c**) Specimen 8 (A+P+15Gl), and (**d**) Specimen 12 (A+P+1GA).

As can be seen from Figure 9a–d, which show the pore size distribution for Specimens 0, 5, 8, and 12, the size of the pores is predominantly below 0.5 mm, with pore sizes most frequently ranging from 0.1 to 0.3 mm. The second group of specimens possess pore sizes with diameters between 0.5 and 1.0 mm, whilst a minority of pores have sizes above 1.0 mm. The pore size distribution trend is very similar for Specimens 0, 5, and 8 and slightly different for Specimen 12 for pore sizes up to 0.4 mm.

### 2.2. FTIR Analysis

**Figure 10:**
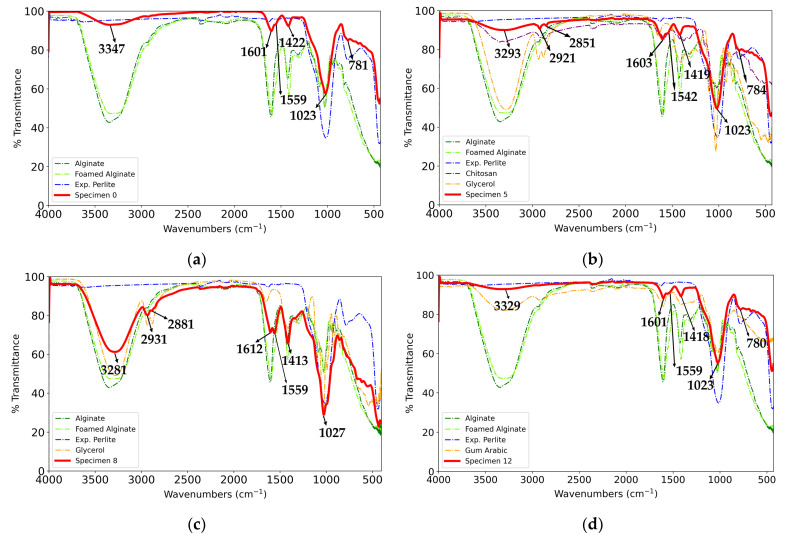
FTIR spectra of specimens and their components: (**a**) Specimen 0, (**b**) Specimen 5, (**c**) Specimen 8, and (**d**) Specimen 12.

The obtained FTIR spectra of selected specimens (Figure 10a–d) indicate the presence of characteristic alginate bands, which is in accordance with the literature data. In addition to these spectra, the spectra of constituents are also given, including the spectrum of alginate without reinforcement and additives that were foamed using the same procedure as that for the specimens. The bands in the range of 3400–3200 cm^−1^ can be assigned to the stretching vibrations of -OH groups, and those in the range of 2921–2931 cm^−1^ can be assigned to the stretching vibrations of aliphatic -CH groups. The bands at 1600–1612 cm^−1^ and 1413–1419 cm^−1^ can be attributed to the asymmetric and symmetric stretching vibrations of carboxylate groups, respectively [64,65,66,67]. The strong large band at 1015 cm^−1^ and the band at 784 cm^−1^ can be assigned to asymmetric and symmetric Si-O-Si (or Si-O-Al) stretching vibrations, respectively, in the expanded perlite [68].

The band at 2851 cm^−1^ in the spectrum of Specimen 5 (Figure 10b) can be assigned to -CH stretching vibrations [69], and the band at 1542 cm^−1^ can be attributed to amide II vibrations in chitosan [70]. The peak at 2881 cm^−1^ in Specimen 8’s spectrum (Figure 10c) can be assigned to -CH stretching vibrations in glycerol [71]. Regarding Specimen 12, as seen in Figure 10d, specific bands of gum arabic [69,72] are in the wavenumber ranges of the specimen’s bands and are not distinctively observable on the spectra of the specimen. The bands at 1559 cm^−1^ for Specimens 0, 8, and 12 can be attributed to the stretching vibrations of the carbonyl group in the acetate ion [73,74].

### 2.3. SEM Analysis

**Figure 11:**
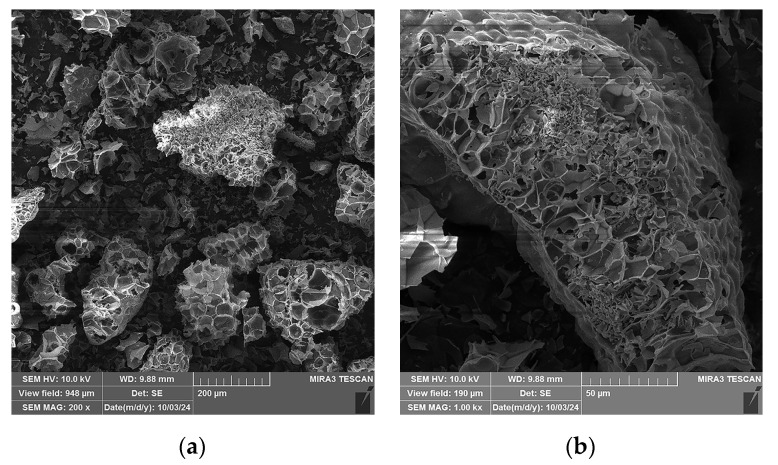
SEM micrographs of the expanded perlite used in this study: (**a**) 200× magnification, (**b**) 1000× magnification.

SEM micrographs of the expanded perlite particles used in this study at different magnifications are presented in Figure 11. A specific expanded perlite structure consisting of pores separated by thin walls is observed (Figure 11a). Some of the pores do not have outer shells, or they are partially broken, as can be seen on the left side of the particle presented in Figure 11b, while other pores are enclosed within the shell walls, which can be observed on the right upper side of the same particle. The central upper part of the presented particle is covered by smaller shell shreds.

**Figure 12:**
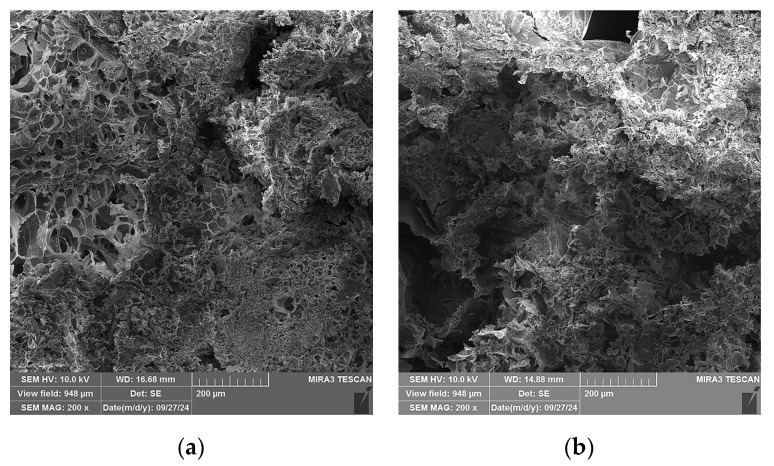

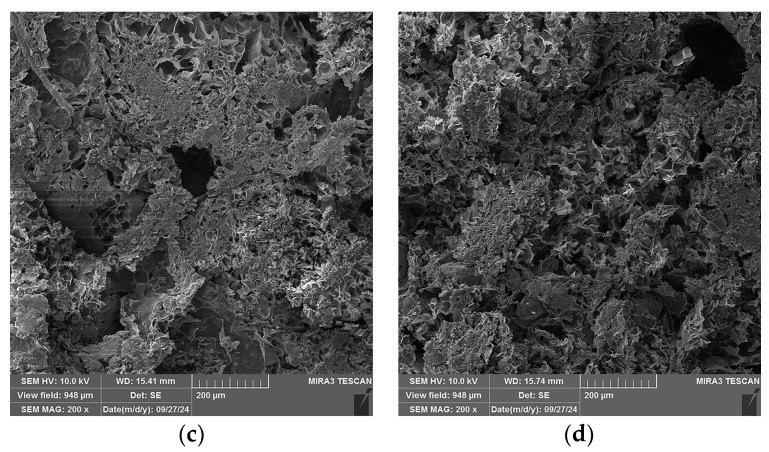
SEM micrographs of selected specimens, 200× magnification: (**a**) Specimen 0 (A+P), (**b**) Specimen 5 (A+P+Ch+5Gl), (**c**) Specimen 8 (A+P+15Gl), (**d**) Specimen 12 (A+P+1GA).

In Figure 12, SEM micrographs of four selected specimens are shown: Specimen 0, containing only A and P and no plasticizers; Specimen 5, containing A, P, Ch, and glycerol plasticizer (1 wt.%); Specimen 8, containing A, P, and glycerol (15 wt.%); and Specimen 9, containing A, P, and gum arabic plasticizer (1 wt.%). A change in the microstructure of the specimens is observed when comparing the micrographs in Figure 12a–d. For Specimen 0 (Figure 12a), segments with expanded perlite particle pores can be clearly observed, whilst for the other three specimens (Figure 12b–d), it can be observed that the pores are mostly covered by alginate layers, with the particles of Specimen 5 (Figure 12b) being covered the most.

**Figure 13:**
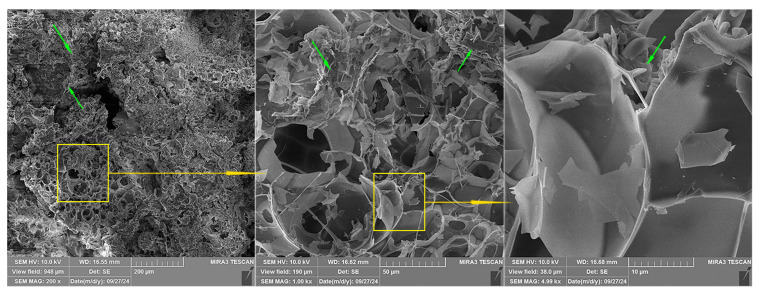
Different magnifications of SEM micrographs of the same area in Specimen 0 From left to right: 200×, 1000×, and 5000× magnification.

Figure 13 displays three magnitudes of magnification for one segment of an expanded perlite particle. For all three magnifications, organic segments can be observed (green arrows).

### 2.4. Brazilian Test

Based on the experimental stress–strain elastic modulus values, obtained via linear regression, the toughness values calculated using Simpson’s rule, along with the calculated tensile strength values, are given in Table 1.

**Figure 14:**
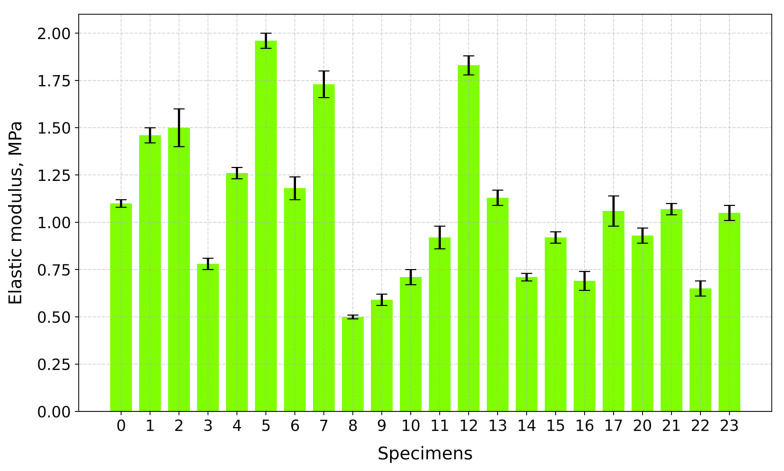
Elastic modulus of tested specimens.

Figure 14 displays the elastic modulus values of the tested specimens. The highest modulus values are reported: Specimen 5 (A+P+Ch+5Gl)—1.96 MPa; Specimen 12 (A+P+1GA)—1.83 MPa; and Specimen 7 (A+P+Ch+IC+5Gl)—1.73 MPa. All of these specimens contain a plasticizer. Some specimens without plasticizers also have higher modulus values: Specimen 2 (A+P+IC)—1.50 MPa—and Specimen 1 (A+P+Ch)—1.46 MPa. Specimens 8 (A+P+15Gl) (0.50 MPa) and 9 (A+P+Ch+15Gl) (0.59 MPa) have the lowest modulus values.

**Figure 15:**
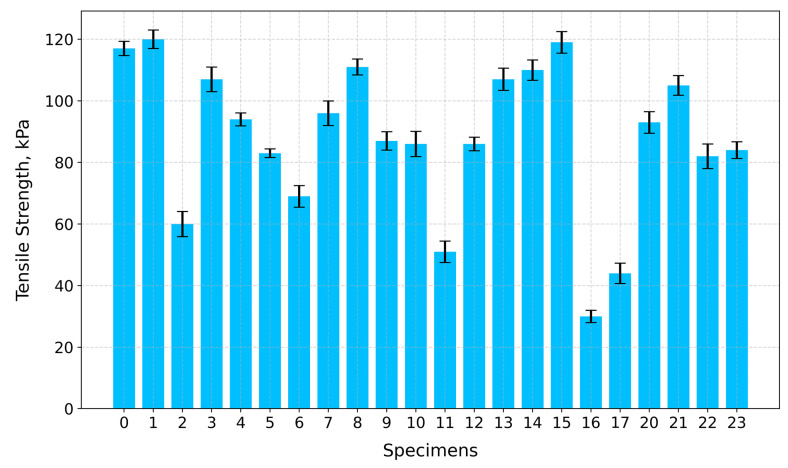
Tensile strength of tested specimens.

Regarding tensile strength (Figure 15), the highest values are for Specimen 1 (A+P+Ch)—120 kPa—Specimen 15 (A+P+5GA)—119 kPa—and Specimen 0 (A+P)—117 kPa—whilst the lowest values are for Specimen 16 (A+P+Ch+IC+1GA)—30 kPa—and Specimen 17 (A+P+Ch+5GA)—44 kPa.

**Figure 16:**
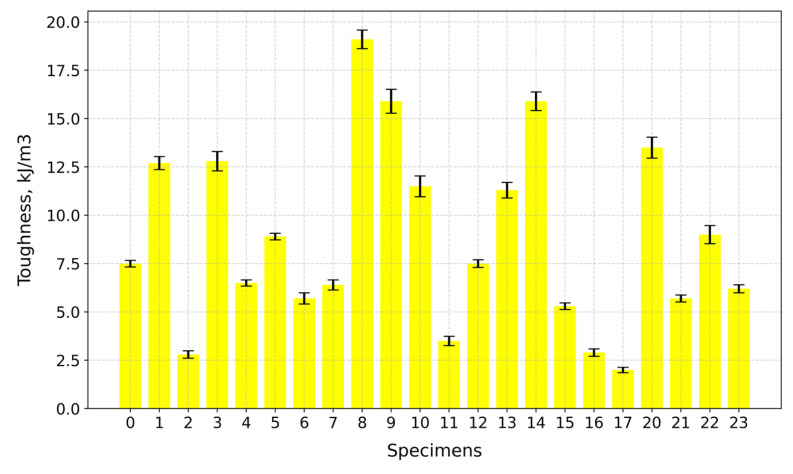
Toughness of tested specimens.

The highest toughness value is observed for Specimen 8 (A+P+15Gl) at 19.1 kJ/m^3^, while the lowest is for Specimen 17 (A+P+Ch+5GA) at 2.0 kJ/m^3^, as can be seen in Figure 16.

When comparing these values on a group (same plasticizer) level, the group with the highest elastic modulus values is the 5 wt.% glycerol plasticizer group, and the group with the highest tensile strength values is the 1 wt.% gum arabic plasticizer group (Specimen 12 excluded), while the 15 wt.% glycerol plasticizer group exhibits the highest toughness values (Specimen 11 excluded).

**Figure 17:**
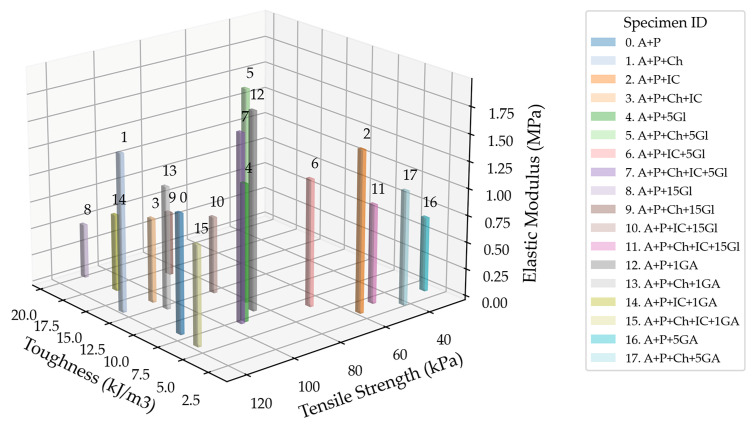
Three-dimensional plot combining the elastic modulus, tensile strength, and toughness value ratios of the tested specimens, excluding the group with the acrylic binder as a plasticizer.

Figure 17 displays the ratio of three different properties: elastic modulus, tensile strength, and toughness. The specimen with the optimal combination of these properties (the highest possible values in a given set of values for a specimen) is Specimen 1 (A+P+Ch). On the other hand, Specimen 16 (A+P+Ch+IC+1GA) has the combination with the lowest values.

Considering the similar behavior of the materials in the studied elastic range during tension and compression loading [75], the obtained elastic modulus values are comparable to the compression modulus values of reinforced aerogel composites that are intended for use as thermal insulation, with values ranging from 1.62 MPa to 3.42 MPa [76]. In addition, the range of elastic modulus values for the tested specimens is also comparable to the values of biocomposite foam materials that are used for cushioning, packaging, and insulation, which range from 0.047 to 2.18 MPa [77].

### 2.5. Water Absorption Test

**Figure 18:**
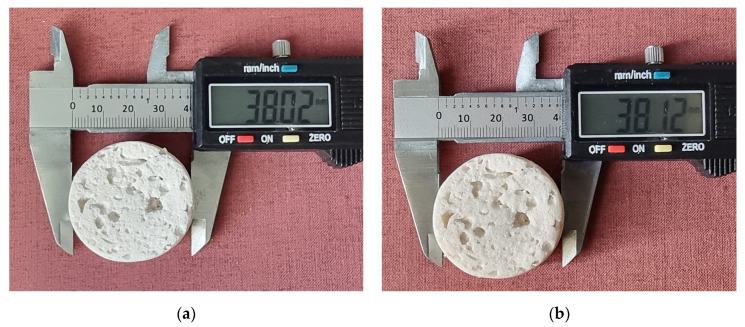
A specimen (**a**) before and (**b**) after water absorption testing.

Figure 18a and Figure 18b show the appearance and dimensions of a specimen before and after testing, respectively. All specimens maintained their dimensional and structural stability. In Table 2, the percentage absorption values for each specimen are given.

The minimal absorption values, with respect to specific plasticizers, are for the 15% glycerol plasticizer group (Specimens 8–11), followed by the acrylic binder group (Specimens 20–23), while the maximum absorptions are observed for the 1% gum arabic group (Specimens 12–15). The lowest water absorption value of 64.8% is for Specimen 8 (A+P+15Gl), and the highest, 205%, is for Specimen 14 (A+P+IC+1GA).

## 3. Conclusions

In this work, the influence of different additives in an alginate-matrix-based expanded-perlite-reinforced composite foam material was investigated. Specimens were prepared using a foaming reaction between acetic acid and sodium bicarbonate, where, in contact with specimen component mixture, the CO_2_ bubbles were released, creating pores in the bulk mass of the material, and were trapped by submerging the specimen in a CaCl_2_ bath. Dried specimens were subjected to the Brazilian test, and based on the determined mechanical properties, the choice of additives for further investigation was narrowed down. The results highlighted the following:

1. Specimens without plasticizers have increased elastic modulus values, as observed in the case of Specimens 1 (1.46 MPa) and 2 (1.50 MPa), compared to Specimen 0 (1.10 MPa).
2. The addition of chitosan improves the elastic modulus, as noted for Specimens 1 (32.7%), 5 (55.5%), 7 (46.6%), 9 (18.0%), 11 (29.6%), and 17 (53.6%), compared to the same specimens without chitosan (0, 4, 6, 8, 10, and 16, respectively).
3. Using different percentages of the same plasticizer affects the composite’s modulus of elasticity, which can be seen by comparing Specimens 4 (with 5% glycerol (1.26 MPa)) and 8 (with 15% glycerol (0.50 MPa)) and Specimens 12 (with 1% gum arabic (1.83 MPa)) and 16 (with 5% gum arabic (0.69 MPa)). From the obtained results, it can be clearly seen that small percentages of plasticizer improve the modulus values, while higher percentages lead to a significant drop. In addition, the modulus of elasticity of specimens with a lower content of glycerol plasticizer (1.26 MPa) also increases with the presence of chitosan (1.96 MPa).
4. The addition of chitosan and/or iota carrageenan in specimens with a lower percentage of gum arabic reduces the elastic modulus (1.83 MPa compared to 1.13 MPa, 0.71 MPa, and 0.92 MPa). On the other hand, when it comes to the tensile strength and toughness, the addition of either chitosan or iota carrageenan leads to an increase (107 kPa and 110 kPa compared to 86 kPa, and 11.3 kJ/m^3^ and 15.9 kJ/m^3^ compared to 7.5 kJ/m^3^, respectively).
5. Adding glycerol plasticizer at higher weight percentages improves the toughness value, as can be seen for Specimens 8 (19.1 kJ/m^3^), 9 (15.9 kJ/m^3^), and 10 (11.5 kJ/m^3^) compared to the specimens with a lower percentage of glycerol (6.5 kJ/m^3^, 8.9 kJ/m^3^, 5.7 kJ/m^3^, and 6.4 kJ/m^3^). Similar results were also observed for gum arabic plasticizer at lower weight percentages; the tensile strength values of Specimens 13 (107 kPa), 14 (110 kPa), and 15 (119 kPa) were higher compared to specimens with higher gum arabic percentages (30 kPa and 44 kPa). The toughness values of Specimens 13 (11.3 kJ/m^3^) and 14 (15.9 kJ/m^3^) were also increased compared to specimens with higher gum arabic percentages (2.9 kJ/m^3^ and 2.0 kJ/m^3^).
6. The toughness increases when plasticizer is added in higher wight percentages; the increase is 193.8%, 78.6% and 101.7% for Specimens 8, 9, 10 (15% glycerol), respectively, compared to the corresponding Specimens 4, 5, 6 (5% glycerol), respectively.
7. In the presence of iota carrageenan, along with gum arabic and chitosan, the toughness is reduced for specimen groups with a higher percentage of glycerol (15.9 kJ/m^3^ and 11.5 kJ/m^3^ compared to 3.5 kJ/m^3^) and specimen groups with a lower percentage of gum arabic (11.3 kJ/m^3^ and 15.9 kJ/m^3^ compared to 5.3 kJ/m^3^).
8. The addition of iota carrageenan should be avoided when gum arabic is present at higher weight percentages, as this leads to shedding (Specimens 18 and 19).
9. The combination of both chitosan and carrageenan, along with plasticizers, should be avoided when plasticizers are present at higher weight percentages, as these specimens in general did not exhibit good mechanical behavior.
10. The specimen possessing the optimal combination of tensile strength, elastic modulus, and toughness, if the criterion for optimality is based on the combination of their highest values, is Specimen 1 (120 kPa, 1.46 MPa, 12.7 kJ/m^3^), while Sample 16 is the weakest (30 kPa, 0.69 MPa, 2.9 kJ/m^3^).
11. Regarding water absorption, a higher percentage of glycerol plasticizer reduces water absorption, which was observed for Specimens 8 (64.8%), 9 (74.8%), 10 (77.0%), and 11 (88.0%) compared to Specimens 4 (114.4%), 5 (164%), 6 (107.7%), and 7 (118.2%), respectively. All specimens maintained their structural stability.

Based on the above findings, the most promising combinations of materials for further investigation and improvement to create alginate–expanded perlite composites materials are as follows: (1) combinations of chitosan with a low percentage glycerol or (2) a low percentage of gum arabic with or without chitosan.

## 4. Materials and Methods

### 4.1. Materials

As a matrix/binder, a commercial pure-grade sodium alginate powder, provided by Würzteufel (Horb am Neckar, Germany), was used. Calcium chloride, for the calcium bath, was also obtained from Würzteufel (Horb am Neckar, Germany), whilst chitosan powder was obtained from Mystic Moments (Sandleheath, UK), glycerol was obtained from Zorka Pharma Hemija (Šabac, Serbia), gum arabic powder was provided by CE Roeper (Hamburg, Germany), and iota carrageenan powder was provided by Special Ingredients (Chesterfield, UK). For the acrylic resin, Acronal A 754, an aqueous dispersion of acrylic copolymer from BASF (Ludwigshafen, Germany), was used. Acetic acid and sodium bicarbonate were obtained from Zorka Pharma Hemija (Šabac, Serbia). Expanded perlite, type P2, with a bulk density of 66 kg/m^3^, was obtained from Termika (Zrenjanin, Serbia). It is composed mainly of SiO_2_ (68–75%) and Al_2_O_3_ (10–12%), and its granulometric content is given in Table 3.

Calcium chloride, CaCl_2_, was dissolved in demineralized water to make a 5 wt.% solution. Sodium alginate was dissolved in demineralized water via gradual addition with constant mechanical mixing at a speed of 3600 rounds/min for 10 min to make a 3 wt.% solution; after mixing, the gel was left to rest until no bubbles remained in the solution. Acetic acid was diluted with demineralized water to obtain a 20 vol.% solution.

### 4.2. Specimen Preparation

Specimens were prepared according to the procedure described below in the text, with compositions (different additives combinations) given in Table 4. Alginate (3 wt.%) and perlite (9 wt.%) were used in all specimens.

All components were added to 3 wt.% sodium alginates one by one in the following order: perlite (P), chitosan (Ch) (if included), iota carrageenan (IC) (if included), glycerol (Gl) (if included), gum arabic (GA) (if included), and acrylic binder (AB) (if included). The initial mass of alginate gel was 20.0 g. Perlite was added to correspond to 9 wt.% of the complete mixture. Further, chitosan was added as 0.95 wt.% of the mixture and iota carrageenan as 0.10 wt.% of the mixture. Glycerol was added at two different concentrations: 5 wt.% and 10 wt.%. Gum arabic was also added at two different concentrations: 1 wt.% and 5 wt.%. Acrylic resin was added to correspond to 10 wt.% of the mixture. After the addition of each component, the mixture was mixed for 5 min to homogenize the added component. Upon adding all components corresponding to the specific specimen from the composition table, 1.0 g of sodium bicarbonate was added to the mixture, without any mixing, covering the surface area of mixture. Then, 20 vol.% acetic acid was poured over the mixture in stoichiometric proportion according to the following equation:

CH_3_COOH + NaHCO_3_ = CH_3_COONa + H_2_O + CO_2_ (1)

The reaction immediately started, and carbon dioxide (CO_2_) was released, and at the same time, the mixture was mechanically mixed using a thin wide spatula at a speed of 90 rounds/min to incorporate the CO_2_ bubbles. After 30 s, the mixture was poured into a cylindrical specimen holder. Any residual mixture left on the specimen cup walls was removed. Then, 5 wt.% CaCl_2_ was gently poured down the walls of the holder up to the top. The cup holder was then closed with a lid and rotated 10 times to enable the CaCl_2_ solution to fully cover all outer surfaces of the specimen. The specimens were left for 24 h to settle. Afterwards, they were left to air-dry for another 24 h and then oven-dried at 30 °C for 18 h. Upon removal from the oven, they were left to cool to room temperature for another 8 h. Then, they were ready for testing. All prepared specimens were structurally stable, except for Specimens 18 (A+P+IC+5GA) and 19 (A+P+Ch+IC+5GA), which displayed dissipating and crumbling effects and lacked structural stability; due to this, they were excluded from testing.

### 4.3. Characterization Methods

Image analysis was performed using free open-source Python programming language and the image processing libraries Matplotlib, NumPy, and OpenCV. Images were first enhanced and then thresholder to obtain segmented images showing the pore shapes of specimens, based on which the calculations of surface pore ratios were performed.

Using a 20 kV emission scanning electron microscope (FE-SEM Mira3 Tescan, Oxford, UK), the samples’ microstructures were examined. Before imaging, a small layer of Au was sputtered on the sample.

A NicoletTM iSTM 10 FT-IR spectrometer (ThermoFisher SCIENTIFIC, Waltham, MA, USA) with Smart iTRTM Attenuated Total Reflectance (ATR) sampling accessories was used to measure the Fourier transform infrared (FTIR) spectra in 20 scan modes in the range of 4000–500 cm^–1^ with a 4 cm^–1^ resolution.

Brazilian tests were performed according to the standard SRPS B.B7.127:2020 [78] using a Texture Analyzer Shimadzu EZ Test LX (Shimadzu, Kyoto, Japan) machine equipped with a 5 kN load cell. Specimens were compressed at a 1 mm/min loading rate. The tensile strength was calculated using Formula (1):

(2) $$\sigma_{t}=\frac{2P}{\pi Dt}$$

where *P* represents the maximum applied load, *D* is the diameter of the disk specimen, and *t* is the thickness of the disk specimen. For each composition, 3 specimens were prepared and tested, and the average test result values were used for further calculations. Based on the test data, a stress–strain diagram was plotted, and the elastic modulus was obtained for each specimen. Tensile properties are the most important properties to analyze when a material is considered for use. Materials in foam form are usually weak with regard to tensile testing, so this method is the harshest way to test material behavior. On the other hand, considering the polymeric binders, under compression, this material tends to be squeezed and to lose its form, so the end of the test is not very clear. Therefore, the tensile properties were tested in the first round of testing.

**Figure 19:**
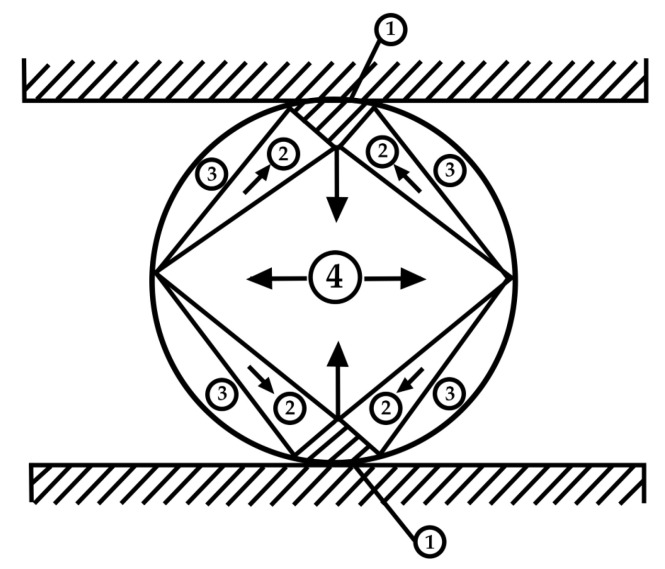
Zones that appear during the tensile test.

Given that the specimens are porous and soft, it is not possible to use standard test tubes to test the tensile strength; thus, specimens were fabricated into cylindrical form with dimensions of 44 × 15 mm. For these specimens, the Brazilian test was chosen for testing the tensile properties, according to Figure 19. The figure shows the zones that appear during the tensile test [78].

Under such stress, four zones are distinguished: 1—zone of triaxial stress; 2—zone of uniaxial stress; 3—neutral zone; and 4—tension zone.

**Figure 20:**
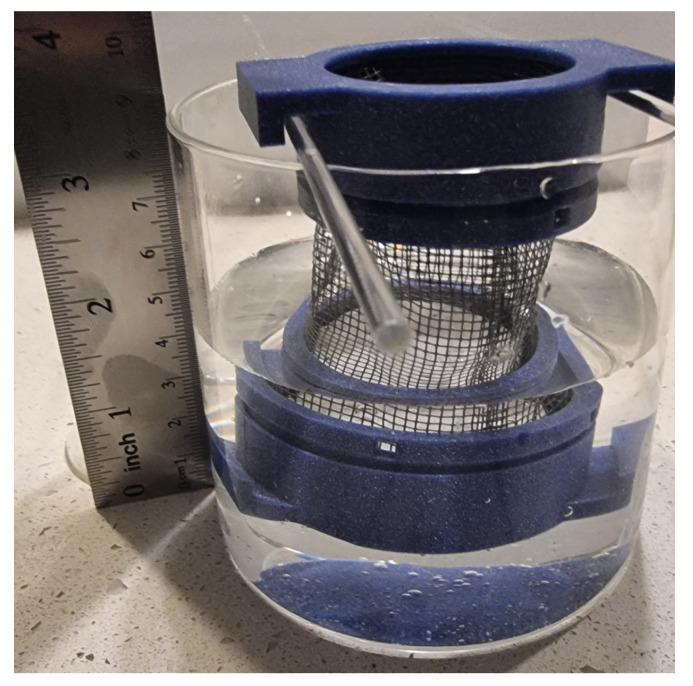
Procedure of submerging specimens in water.

The water absorption test was conducted according to the ASTM standard D570 [79]. Since the material we are investigating is porous, they were immersed for 2 h. First, the specimens were dried to a constant mass (in an oven at 30 °C), after which their mass and diameter were measured. Then, the specimens were submerged in distilled water for 2 h (Figure 20). After this, the surface moisture was removed with a cloth, and their mass and diameter were measured again. Water absorption was calculated using the following formula [79]:

Water absorption = 100 × (wet weight − dry weight)/dry weight(3)

## Figures and Tables

**Table 1:** Tensile strength, elastic modulus, toughness, largest pore diameter, surface pore ratio, density values, and diameter shrinkage of tested specimens.

Specimen	Tensile Strength, kPa	Elastic Modulus, MPa	Toughness, kJ/m^3^	Mean Surface Pore Ratio, %	Density (Disk), g/cm^3^	Diameter Shrinkage After Drying, %
0	117	1.10	7.5	24.8	0.118	14.3
1	120	1.46	12.7	35.0	0.141	18.0
2	60	1.50	2.8	35.8	0.118	14.3
3	107	0.78	12.8	30.2	0.153	17.3
4	94	1.26	6.5	35.5	0.161	16.6
5	83	1.96	8.9	40.0	0.128	14.8
6	69	1.18	5.7	38.7	0.127	16.8
7	96	1.73	6.4	34.3	0.155	13.0
8	111	0.50	19.1	35.1	0.199	17.5
9	87	0.59	15.9	34.9	0.181	15.5
10	86	0.71	11.5	33.1	0.193	15.5
11	51	0.92	3.5	34.2	0.170	12.3
12	86	1.83	7.5	35.3	0.106	12.7
13	107	1.13	11.3	34.1	0.119	11.4
14	110	0.71	15.9	44.3	0.104	14.1
15	119	0.92	5.3	36.7	0.151	14.1
16	30	0.69	2.9	46.3	0.119	10.9
17	44	1.06	2.0	45.7	0.112	12.4
20	93	0.93	13.5	40.0	0.117	11.6
21	105	1.07	5.7	39.9	0.114	11.4
22	82	0.65	9.0	39.6	0.127	14.3
23	84	1.05	6.2	45.2	0.139	14.8

**Table 2:** Water absorption values of specimens.

Specimen	Water Absorption, %	Specimen	Water Absorption, %
0	142.9	11	80.8
1	112.3	12	156.8
2	163.8	13	137.1
3	110.5	14	205.0
4	114.4	15	144.4
5	164.0	16	103.9
6	107.7	17	197.4
7	118.2	20	101.6
8	64.8	21	94.4
9	74.8	22	96.8
10	77.0	23	84.2

**Table 3:** Granulometric distribution of P2-type perlite.

Parameter	Sieve Diameter [mm]	Average Value
	+1.000	11.4
Granulometric content	−1.000 + 0.315	52.8
	−0.315 + 0.200	12.7
	−0.200	23.1

**Table 4:** Overview of specimen compositions.

Specimen No.	Alginate 3 wt.% Perlite 9 wt.%	Chitosan 0.95 wt.%	Iota Carrageenan 0.1 wt.%	Glycerol		Gum Arabic		Acrylic Binder 10 wt.%
				5 wt.%	15 wt.%	1 wt.%	5 wt.%	
0	●	x	x	x	x	x	x	x
1	●	●	x	x	x	x	x	x
2	●	x	●	x	x	x	x	x
3	●	●	●	x	x	x	x	x
4	●	x	x	●	x	x	x	x
5	●	●	x	●	x	x	x	x
6	●	x	●	●	x	x	x	x
7	●	●	●	●	x	x	x	x
8	●	x	x	x	●	x	x	x
9	●	●	x	x	●	x	x	x
10	●	x	●	x	●	x	x	x
11	●	●	●	x	●	x	x	x
12	●	x	x	x	x	●	x	x
13	●	●	x	x	x	●	x	x
14	●	x	●	x	x	●	x	x
15	●	●	●	x	x	●	x	x
16	●	x	x	x	x	x	●	x
17	●	●	x	x	x	x	●	x
18	●	x	●	x	x	x	●	x
19	●	●	●	x	x	x	●	x
20	●	x	x	x	x	x	x	●
21	●	●	x	x	x	x	x	●
22	●	x	●	x	x	x	x	●
23	●	●	●	x	x	x	x	●

●: present in the specimen; x: not present in the specimen.

## Data Availability

The data presented in this study are available on request from the corresponding author or co-authors. The data are not publicly available.

## References

[1] Hecht H., Srebnik S. (2016). Structural Characterization of Sodium Alginate and Calcium Alginate. Biomacromolecules.

[2] Ching S.H., Bansal N., Bhandari B. (2017). Alginate Gel Particles–A Review of Production Techniques and Physical Properties. Crit. Rev. Food Sci. Nutr..

[3] Soares J.P., Santos J.E., Chierice G.O., Cavalheiro E.T.G. (2004). Thermal Behavior of Alginic Acid and Its Sodium Salt. Eclét. Quím..

[4] Gheorghita Puscaselu R., Lobiuc A., Dimian M., Covasa M. (2020). Alginate: From Food Industry to Biomedical Applications and Management of Metabolic Disorders. Polymers.

[5] Hurtado A., Aljabali A.A.A., Mishra V., Tambuwala M.M., Serrano-Aroca Á. (2022). Alginate: Enhancement Strategies for Advanced Applications. Int. J. Mol. Sci..

[6] Hu C., Lu W., Mata A., Nishinari K., Fang Y. (2021). Ions-Induced Gelation of Alginate: Mechanisms and Applications. Int. J. Biol. Macromol..

[7] Grant G.T., Morris E.R., Rees D.A., Smith P.J.C., Thom D. (1973). Biological Interactions between Polysaccharides and Divalent Cations: The Egg-box Model. FEBS Lett..

[8] Wei Q., Zhou J., An Y., Li M., Zhang J., Yang S. (2023). Modification, 3D Printing Process and Application of Sodium Alginate Based Hydrogels in Soft Tissue Engineering: A Review. Int. J. Biol. Macromol..

[9] Leonardo M., Prajatelistia E., Judawisastra H. (2022). Alginate-Based Bioink for Organoid 3D Bioprinting: A Review. Bioprinting.

[10] Abourehab M.A.S., Rajendran R.R., Singh A., Pramanik S., Shrivastav P., Ansari M.J., Manne R., Amaral L.S., Deepak A. (2022). Alginate as a Promising Biopolymer in Drug Delivery and Wound Healing: A Review of the State-of-the-Art. Int. J. Mol. Sci..

[11] Cervino G., Fiorillo L., Herford A.S., Laino L., Troiano G., Amoroso G., Crimi S., Matarese M., D’Amico C., Nastro Siniscalchi E. (2018). Alginate Materials and Dental Impression Technique: A Current State of the Art and Application to Dental Practice. Mar. Drugs.

[12] Beni A.A., Esmaeili A. (2020). Biosorption, an Efficient Method for Removing Heavy Metals from Industrial Effluents: A Review. Environ. Technol. Innov..

[13] Akter N., Akter N., Pervin M., Repon M.R. (2023). The Influence of Mixed Thickeners on Printing over Lyocell Knitted Fabric. Heliyon.

[14] Xu Y.-J., Qu L.-Y., Liu Y., Zhu P. (2021). An Overview of Alginates as Flame-Retardant Materials: Pyrolysis Behaviors, Flame Retardancy, and Applications. Carbohydr. Polym..

[15] Jiang Y., Pang X., Deng Y., Sun X., Zhao X., Xu P., Shao P., Zhang L., Li Q., Li Z. (2019). An Alginate Hybrid Sponge with High Thermal Stability: Its Flame Retardant Properties and Mechanism. Polymers.

[16] Xu H., Liu C., Guo W., Li N., Chen Y., Meng X., Zhai M., Zhang S., Wang Z. (2024). Sodium Alginate/Al_2_O_3_ Fiber Nanocomposite Aerogel with Thermal Insulation and Flame Retardancy Properties. Chem. Eng. J..

[17] Ahmad Raus R., Wan Nawawi W.M.F., Nasaruddin R.R. (2021). Alginate and Alginate Composites for Biomedical Applications. Asian J. Pharm. Sci..

[18] Lee K.Y., Mooney D.J. (2012). Alginate: Properties and Biomedical Applications. Prog. Polym. Sci..

[19] Eslami Z., Elkoun S., Robert M., Adjallé K. (2023). A Review of the Effect of Plasticizers on the Physical and Mechanical Properties of Alginate-Based Films. Molecules.

[20] Mohammed A., Gaduan A., Chaitram P., Pooran A., Lee K.-Y., Ward K. (2023). Sargassum Inspired, Optimized Calcium Alginate Bioplastic Composites for Food Packaging. Food Hydrocoll..

[21] Lacoste C., El Hage R., Bergeret A., Corn S., Lacroix P. (2018). Sodium Alginate Adhesives as Binders in Wood Fibers/Textile Waste Fibers Biocomposites for Building Insulation. Carbohydr. Polym..

[22] Zhou Y., Trabelsi A., El Mankibi M. Development and Characterization of Thermal Insulation Materials Based on Rice Straw and Natural Binder. Proceedings of the CLIMA 2022 The 14th REHVA HVAC World Congress.

[23] Palumbo M., Formosa J., Lacasta A.M. (2015). Thermal Degradation and Fire Behaviour of Thermal Insulation Materials Based on Food Crop By-Products. Constr. Build. Mater..

[24] Gurgel M., Vieira A., Altenhofen M., Oliveira L., Beppu M.M. (2011). Natural-Based Plasticizers and Biopolymer Films: A Review. Eur. Polym. J..

[25] da Silva M.A., Bierhalz A.C.K., Kieckbusch T.G. (2009). Alginate and Pectin Composite Films Crosslinked with Ca^2+^ Ions: Effect of the Plasticizer Concentration. Carbohydr. Polym..

[26] Wang X., Zhang H., Zhang X., Shen C., Liu M., Liu S., Han Y., He T. (2024). A Comparison Study on Effects of Polyglycerols on Physical Properties of Alginate Films. Int. J. Biol. Macromol..

[27] Tong Q., Xiao Q., Lim L. (2013). Effects of Glycerol, Sorbitol, Xylitol and Fructose Plasticisers on Mechanical and Moisture Barrier Properties of Pullulan–Alginate–Carboxymethylcellulose Blend Films. Int. J. Food Sci. Technol..

[28] Ben Z.Y., Samsudin H., Yhaya M.F. (2022). Glycerol: Its Properties, Polymer Synthesis, and Applications in Starch Based Films. Eur. Polym. J..

[29] Wolfson A., Dlugy C., Shotland Y. (2007). Glycerol as a Green Solvent for High Product Yields and Selectivities. Environ. Chem. Lett..

[30] Prasad N., Thombare N., Sharma S.C., Kumar S. (2022). Gum Arabic—A Versatile Natural Gum: A Review on Production, Processing, Properties and Applications. Ind. Crops Prod..

[31] Feraru A., Tóth Z.-R., Mureșan-Pop M., Baia M., Gyulavári T., Páll E., Turcu R.V.F., Magyari K., Baia L. (2023). Anionic Polysaccharide Cryogels: Interaction and In Vitro Behavior of Alginate–Gum Arabic Composites. Polymers.

[32] Tsai F.-H., Kitamura Y., Kokawa M. (2017). Effect of Gum Arabic-Modified Alginate on Physicochemical Properties, Release Kinetics, and Storage Stability of Liquid-Core Hydrogel Beads. Carbohydr. Polym..

[33] Zakka W. (2019). Suitability of Gum Arabic as a Plasticizer in Self-Compacting Concrete Compacting Concrete Compacting Concrete: Fresh Concrete Fresh Concrete Properties. Int. J. Environ. Stud. Saf. Res..

[34] Li M., Li H., Li X., Zhu H., Xu Z., Liu L., Ma J., Zhang M. (2017). A Bioinspired Alginate-Gum Arabic Hydrogel with Micro-/Nanoscale Structures for Controlled Drug Release in Chronic Wound Healing. ACS Appl. Mater. Interfaces.

[35] Jiménez-Gómez C.P., Cecilia J.A. (2020). Chitosan: A Natural Biopolymer with a Wide and Varied Range of Applications. Molecules.

[36] Ramdhan T., Ching S.H., Prakash S., Bhandari B. (2020). Physical and Mechanical Properties of Alginate Based Composite Gels. Trends Food Sci. Technol..

[37] Kim K., Cheng J., Liu Q., Wu X.Y., Sun Y. (2010). Investigation of Mechanical Properties of Soft Hydrogel Microcapsules in Relation to Protein Delivery Using a MEMS Force Sensor. J. Biomed. Mater. Res. Part A.

[38] Baruch L., Machluf M. (2006). Alginate–Chitosan Complex Coacervation for Cell Encapsulation: Effect on Mechanical Properties and on Long-term Viability. Biopolymers.

[39] Segale L., Giovannelli L., Mannina P., Pattarino F. (2016). Calcium Alginate and Calcium Alginate-Chitosan Beads Containing Celecoxib Solubilized in a Self-Emulsifying Phase. Scientifica.

[40] Bui V.T.N.T., Nguyen B.T., Nicolai T., Renou F. (2019). Mixed Iota and Kappa Carrageenan Gels in the Presence of Both Calcium and Potassium Ions. Carbohydr. Polym..

[41] Abdul Khalil H.P.S., Tye Y.Y., Saurabh C.K., Leh C.P., Lai T.K., Chong E.W.N., Nurul Fazita M.R., Mohd Hafiidz J., Banerjee A., Syakir M.I. (2017). Biodegradable Polymer Films from Seaweed Polysaccharides: A Review on Cellulose as a Reinforcement Material. Express Polym. Lett..

[42] Zujovic Z., Wheelwright W.V.K., Kilmartin P.A., Hanna J.V., Cooney R.P. (2018). Structural Investigations of Perlite and Expanded Perlite Using 1H, 27Al and 29Si Solid-State NMR. Ceram. Int..

[43] Bush A.L. (2001). Construction Materials: Lightweight Aggregates. Encyclopedia of Materials: Science and Technology.

[44] Berge B. (2009). The Ecology of Building Materials.

[45] Rashad A.M. (2016). A Synopsis about Perlite as Building Material—A Best Practice Guide for Civil Engineer. Constr. Build. Mater..

[46] Roulia M., Chassapis K., Kapoutsis J.A., Kamitsos E.I., Savvidis T. (2006). Influence of Thermal Treatment on the Water Release and the Glassy Structure of Perlite. J. Mater. Sci..

[47] Arifuzzaman M., Kim H.S. (2015). Novel Mechanical Behaviour of Perlite/Sodium Silicate Composites. Constr. Build. Mater..

[48] Shastri D., Kim H.S. (2014). A New Consolidation Process for Expanded Perlite Particles. Constr. Build. Mater..

[49] Guo Y., Tian Q., Lu H., Qu N., Chen L., Zhang Y., Xiao C., Hasi Q. (2022). An Expanded Perlite-Based Aerogel with Oil-Repellent Properties for Efficient Solar Evaporation in Oil-bearing Wastewater. ChemistrySelect.

[50] Türe H., Terzioğlu K., Tunca E. (2017). Characterization of Alginate/Perlite Particles. Süleyman Demirel Üniversitesi Fen Bilim. Enstitüsü Derg..

[51] Parlayici Ş. (2019). Alginate-Coated Perlite Beads for the Efficient Removal of Methylene Blue, Malachite Green, and Methyl Violet from Aqueous Solutions: Kinetic, Thermodynamic, and Equilibrium Studies. J. Anal. Sci. Technol..

[52] Liffourrena A.S., Lucchesi G.I. (2018). Alginate-Perlite Encapsulated Pseudomonas Putida A (ATCC 12633) Cells: Preparation, Characterization and Potential Use as Plant Inoculants. J. Biotechnol..

[53] Chegeni M., Mehri M., Shokri Rozbahani Z. (2023). Cuminum Cyminum L Encapsulaton on Perlite/Calcium Alginate/Single Walled Carbon Nanotubes -Glucose Composite: Fabricaton, Characterizaton, Antbacterial and Antfungal Propertes. Nanochemistry Res..

[54] Iglesias I., Acosta B., Yu R., Ruiz G., Aineto M., Acosta A. (2011). Estudio de Caracterización Mecánica de Probetas Cerámicas a Partir de Una Adaptación Del Ensayo Brasileño. Mater. Constr..

[55] Elghazel A., Taktak R., Bouaziz J. (2015). Determination of Elastic Modulus, Tensile Strength and Fracture Toughness of Bioceramics Using the Flattened Brazilian Disc Specimen: Analytical and Numerical Results. Ceram. Int..

[56] Carmona S., Aguado A. (2012). New Model for the Indirect Determination of the Tensile Stress–Strain Curve of Concrete by Means of the Brazilian Test. Mater. Struct..

[57] Huang Z., Zhang Y., Li Y., Zhang D., Yang T., Sui Z. (2021). Determining Tensile Strength of Rock by the Direct Tensile, Brazilian Splitting, and Three-Point Bending Methods: A Comparative Study. Adv. Civ. Eng..

[58] Price H.L., Murray K.H. (1973). Finite Element Analysis of the Diametral Test of Polymer Moldings. J. Eng. Mater. Technol..

[59] Martínez-López M., Martínez-Barrera G., Nunes L.C.S., Reis J.M.L., da Costa Mattos H.S. (2016). Mixed Mode Fracture Analysis in a Polymer Mortar Using the Brazilian Disk Test. Eng. Fract. Mech..

[60] Vuksanović M., Tomić N., Algellai A., Balanč B., Radovanović Ž., Trifunović D., Jančić-Heinemann R. (2019). Examination of Mechanical Properties of Acrilyc Composite Materials with Different Al_2_O_3_ Reinforcement by Brazil Test. Tehnika.

[61] Al-Allaq A.A., Kashan J.S., El-Wakad M.T., Soliman A.M. (2021). The Bio-Composites (Hydroxyapatite/High-Density Polyethylene) Materials Rein Forced with Multi-Walled Carbon Nanotubes for Bone Tissue Repair. J. Ceram. Process. Res..

[62] Deniz Akin I., Likos W.J. (2017). Brazilian Tensile Strength Testing of Compacted Clay. Geotech. Test. J..

[63] Gaspar T.A.V., Jacobsz S.W. (2021). Brazilian Tensile Strength Test Conducted on Ductile Unsaturated Soil Samples. Geotech. Test. J..

[64] Larosa C., Salerno M., de Lima J.S., Merijs Meri R., da Silva M.F., de Carvalho L.B., Converti A. (2018). Characterisation of Bare and Tannase-Loaded Calcium Alginate Beads by Microscopic, Thermogravimetric, FTIR and XRD Analyses. Int. J. Biol. Macromol..

[65] Fajardo A.R., Silva M.B., Lopes L.C., Piai J.F., Rubira A.F., Muniz E.C. (2012). Hydrogel Based on an Alginate–Ca^2+^/Chondroitin Sulfate Matrix as a Potential Colon-Specific Drug Delivery System. RSC Adv..

[66] Paraskevopoulou P., Gurikov P., Raptopoulos G., Chriti D., Papastergiou M., Kypritidou Z., Skounakis V., Argyraki A. (2018). Strategies toward Catalytic Biopolymers: Incorporation of Tungsten in Alginate Aerogels. Polyhedron.

[67] Gómez-Ordóñez E., Rupérez P. (2011). FTIR-ATR Spectroscopy as a Tool for Polysaccharide Identification in Edible Brown and Red Seaweeds. Food Hydrocoll..

[68] Çelen U., Balçik Tamer Y., Berber H. (2024). The Potential Use of Natural Expanded Perlite as a Flame Retardant Additive for Acrylonitrile-butadiene-styrene Based Composites. J. Vinyl Addit. Technol..

[69] Appolonia Ibekwe C., Modupe Oyatogun G., Ayodeji Esan T., Michael Oluwasegun K. (2017). Synthesis and Characterization of Chitosan/Gum Arabic Nanoparticles for Bone Regeneration. Am. J. Mater. Sci. Eng..

[70] Ramli R.H., Fhong Soon C., Mohd Rus A.Z. (2016). Synthesis of Chitosan /Alginate/ Silver Nanoparticles Hydrogel Scaffold. MATEC Web Conf..

[71] Gawad R., Fellner V. (2019). Evaluation of Glycerol Encapsulated with Alginate and Alginate-Chitosan Polymers in Gut Environment and Its Resistance to Rumen Microbial Degradation. Asian-Australas. J. Anim. Sci..

[72] Sowunmi A., Orodu O., Efeovbokhan V., Ogundare S. (2020). Comparative Dataset on the Characterization of Natural Polymers and Nanocomposites for Enhanced Oil Recovery. Data Br..

[73] Nityananda Agasti N.K.K. (2014). One Pot Synthesis of Crystalline Silver Nanoparticles. Am. J. Nanomater..

[74] Ngo A.N., Ezoulin M.J.M., Murowchick J.B., Gounev A.D., Youan B.-B.C. (2016). Sodium Acetate Coated Tenofovir-Loaded Chitosan Nanoparticles for Improved Physico-Chemical Properties. Pharm. Res..

[75] Beer F.P., Johnston E.R., DeWolf J.T., Mazurek D.F. (2012). Mechanics of Materials.

[76] Shi W., Wan M., Tang Y., Chen W. (2024). Ceramic Fiber-Reinforced Polyimide Aerogel Composites with Improved Shape Stability against Shrinkage. Gels.

[77] Miranda-Valdez I.Y., Coffeng S., Zhou Y., Viitanen L., Hu X., Jannuzzi L., Puisto A., Kostiainen M.A., Mäkinen T., Koivisto J. (2023). Foam-Formed Biocomposites Based on Cellulose Products and Lignin. Cellulose.

[78] (2020). Rock Mechanics—Testing of Physical and Mechanical Properties—Method for the Determination of Tensile Strength Limit—Indirect Method.

[79] (2018). Standard Test Method for Water Absorption of Plastics.

